# Influence of facial cooling on carotid body tonic activity and sensitivity

**DOI:** 10.1113/EP093205

**Published:** 2026-02-02

**Authors:** Robyn Morley, Liam D. Corr, Elliott J. Jenkins, Joseph A. Killick, Travis D. Gibbons, Joshua C. Tremblay

**Affiliations:** ^1^ School of Medicine Cardiff University Cardiff UK; ^2^ Cardiff School of Sport and Health Sciences Cardiff Metropolitan University Cardiff UK; ^3^ Department of Biological Sciences Northern Arizona University Flagstaff Arizona USA

**Keywords:** chemoreflex, cold shock response, hyperoxia, hyperventilation, hypocapnia, hypoxic ventilatory response, trigeminal nerve

## Abstract

Facial cooling can increase ventilation and augment the hypoxic ventilatory response. Whole body cooling increases both carotid body tonic activity and sensitivity; however, whether isolated facial cooling induces similar carotid body hyperexcitability was unknown. We investigated whether facial cooling alters carotid body function by assessing tonic activity and hypoxic sensitivity. Fourteen healthy adults (11 M/3 F; age 26 ± 4 years) completed a counterbalanced, crossover study involving transient hyperoxia and poikilocapnic hypoxia (9.5% O_2_) under thermoneutral (facial temperature: 34.2 ± 1.2°C) and facial cooling (19.4 ± 3.3°C) conditions. Carotid body tonic activity was inferred from the ventilatory suppression during transient hyperoxia. Sensitivity was assessed via the change in end‐tidal CO_2_ ($P_{\mathrm{ETC}O_{2}}$) relative to oxygen saturation ($S_{pO_{2}}$) during hypoxia. Facial cooling induced hyperventilation, evidenced by reduced $P_{\mathrm{ETC}O_{2}}$ (35 ± 8 vs. 41 ± 3 mmHg; *P* = 0.008), and elevated ventilatory equivalent for CO_2_ production (28 ± 6 vs. 23 ± 2; *P* = 0.02). Carotid body tonic activity did not differ between facial cooling and thermoneutral conditions, but carotid body sensitivity was reduced during facial cooling (0.20 ± 0.14 vs. 0.28 ± 0.13 mmHg/%; *P* = 0.044). The reduction in $P_{\mathrm{ETC}O_{2}}$ experienced during facial cooling correlated with enhanced carotid body tonic activity (*R* ^2^ = 0.39, *P* = 0.022) and reduced sensitivity (*R* ^2^ = 0.33, *P* = 0.03). Collectively, facial cooling induces hyperventilation and the attendant hypocapnia reduces carotid body sensitivity. Although this hyperventilation is related to carotid body tonic activity, facial cooling likely produces a cold shock response that stimulates ventilation separately from the carotid body. These findings offer new insights on the interaction between stimuli relevant to outdoor activities in cold environments (e.g., snow shovelling, mountaineering, cold water swimming) and carotid body function.

## Abstract (highlights)

- **What is the central question of this study?**
  Does facial cooling alter carotid body tonic activity or sensitivity?
- **What is the main finding and its importance?**
  Facial cooling induces hyperventilation and reduces carotid body sensitivity. Although the hyperventilation observed with facial cooling is related to carotid body tonic activity, this hyperventilation is likely mediated independently from the carotid body. Our findings advance our understanding of how the environment interacts with the carotid body to influence ventilation.

## 1 INTRODUCTION

Facial exposure to cold air or water is a common occurrence in many natural and occupational environments, as reflected by the high frequency of frostbite on facial regions (Harirchi et al., 2005; Hassi & Mäkinen, 2000; Lehmuskallio et al., 1995). Moreover, isolated facial cooling may contribute to the elevated risk of cardiovascular events associated with cold‐weather activities, such as snow shovelling (Auger et al., 2017; Nichols et al., 2012; Polcaro‐Pichet et al., 2019). Facial cooling stimulates the trigeminal nerve, increasing sympathetic nerve activity (Fagius & Sundlöf, 1986; Fisher et al., 2015; Heindl et al., 2004). This stimulation often provokes hyperventilation (Jay et al., 2007; Stewart et al., 1998), but not always (Mukhtar & Patrick, 1986). Furthermore, a link between trigeminal stimulation and the peripheral chemoreflex has been implicated, as Argacha et al. (2008) demonstrated that facial cooling enhanced the hypoxic ventilatory response. Although the mechanisms are unclear, elevated sympathetic drive causes carotid body hyperexcitability (tonicity and sensitivity) (reviewed in Brognara et al., 2021). However, it is uncertain whether facial cooling alters carotid body tonic activity, a key contributor to baseline ventilatory drive or its sensitivity to hypoxia.

Carotid body tonic activity can be assessed as the ventilatory suppression induced by transient exposures to hyperoxia (Bernards et al., 1966; Gibbons et al., 2022b; Prasad et al., 2020). This causes a rapid rise in the end tidal partial pressure of oxygen ($P_{\mathrm{ET}O_{2}}$) to >250 mmHg, effectively silencing carotid sinus activity (Lahiri et al., 1987). A larger decrease in ventilation during the hyperoxia indicates heightened carotid body tonicity. In humans, increases in carotid body tonic activity occur during whole body heating, cooling and during exercise (Gibbons et al., 2022a, 2022b). Given the profound autonomic response to trigeminal nerve activation, we speculated that facial cooling amplifies carotid body tonic activity and sensitivity.

To examine the influence of facial cooling on carotid body function, we assessed: (i) tonic activity using transient hyperoxia, and (ii) sensitivity using poikilocapnic hypoxia, comparing responses with and without facial cooling in a randomised, counterbalanced, crossover design. We hypothesised that facial cooling would increase both carotid body tonic activity (as indexed by greater ventilatory suppression during hyperoxia) and carotid body sensitivity (as indicated by a stronger ventilatory response to poikilocapnic hypoxia).

## 2 METHODS

### 2.1 Ethical approval

Ethical approval was obtained from the Cardiff Metropolitan University Natural Sciences Panel (Sta‐10786) and conformed to the standard set by the *Declaration of Helsinki*, except for registration in a database. All participants provided written informed consent prior to participation.

### 2.2 Participants

We calculated that a sample size of six was required to detect an effect size of 1.45 (*d_z_*) based on the increase in carotid body tonic activity during whole body cooling (Gibbons et al., 2022b). However, due to the localised instead of whole‐body cooling, we anticipated a smaller effect size and therefore, recruited 15 participants, in line with Argacha et al. (2008). Inclusion criteria included being between 18 and 45 years, having a body mass index (BMI) of <30 kg/m^2^ and being free from overt cardiovascular, neurological or respiratory disease or trigeminal nerve injury. Exclusion criteria were a history of cardiovascular disease, taking cardiovascular‐acting medications, pregnancy, smoking/vaping, currently experiencing symptoms of a cold/illness, or a history of migraine or frequent headaches. One participant was unable to complete the protocol due to discomfort; therefore, the sample size and all data presented are based on 14 participants (11 M/3 F, age: 26 ± 4 years, BMI: 23.5 ± 3.4 kg/m^2^) unless otherwise stated.

### 2.3 Experimental protocol

**FIGURE 1:**
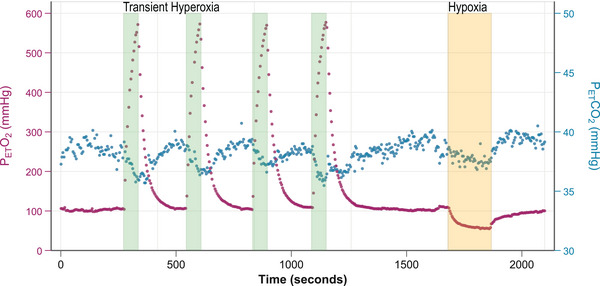
Representative breath‐by‐breath end‐tidal CO_2_ ($P_{\mathrm{ETC}O_{2}}$) and end‐tidal O_2_ ($P_{\mathrm{ET}O_{2}}$) trace of the experimental protocol (single trial). Carotid body tonic activity was assessed as the average reduction in minute ventilation across four transient hyperoxia trials. Carotid body sensitivity was assessed as the reduction in $P_{\mathrm{ETC}O_{2}}$ per reduction in oxygen saturation in the final 30 s of poikilocapnic hypoxia (9.5% O_2_). Note that the *reduction* in $P_{\mathrm{ETC}O_{2}}$ during hyperoxia is likely due to compromised CO_2_ measures that arise when the gas analysers in the metabolic cart are flushed with 100% O_2_.

The study used a single visit, crossover design with counterbalancing (but not randomization); seven participants started with facial cooling, and seven started with the thermoneutral condition. Participants were asked to adhere to their normal daily routine and refrain from strenuous activity on the day of their testing. After measuring height and body mass, participants rested supine for 20 min during instrumentation. Participants were outfitted for cardiorespiratory and thermometry monitoring and then familiarised with breathing on a mouthpiece with a nose clip to optimise the placement of the three‐way valve. Thereafter, ear defenders and an eye cover were applied to minimise external stimuli. Participants breathed room air on the mouthpiece for 3–5 min. After establishing this baseline, participants completed a modified Dejours test. This involved 1 min of breathing 100% O_2_ from a Douglas bag followed by at least 3 min of room air breathing (or until $P_{\mathrm{ET}O_{2}}$ returned to baseline), repeated four times. Following the last hyperoxia cycle, and after $P_{\mathrm{ET}O_{2}}$ returned to baseline, participants breathed one further minute of room air before being switched onto a hypoxic Douglas bag (9.5% O_2_) for a final 3 min before resuming breathing room air. A minimum 20‐min washout was provided before starting the next condition. A representative trace over the course of one trial is presented in Figure 1.

#### 2.3.1 Facial cooling

During the facial cooling condition, a gel ice face mask and eye covers (Cooling gel face set, Medi Grade, Bridlington, UK), direct from a freezer, covered the entire face and eyes. Supplementary gel ice packs were applied over the cheeks to help maintain skin contact. We ensured that skin temperature remained below 28°C throughout the trials to sustain cold‐sensitive trigeminal ganglion neuron activation (de la Peña et al., 2005; McKemy et al., 2002). To maintain facial cooling, the face mask was replaced between the hyperoxia and hypoxia tests in three participants.

### 2.4 Experimental measurements

#### 2.4.1 Cardiorespiratory measurements and thermometry

Participants were instrumented with a lead‐II electrocardiogram (Dual Bio Amp, FE232, ADInstruments, Dunedin, New Zealand) for heart rate, a finger non‐invasive beat‐to‐beat blood pressure monitor (Finometer Pro, Finapres Medical Systems B.V., Enschede, Netherlands), a pulse oximeter (Radical‐7 Pulse CO‐Oximeter, Masimo, Irvine, CA, USA) and a thermistor taped to their cheek to collect facial temperature. These data were continuously sampled at 1000/s using an analog‐to‐digital data acquisition system (PowerLab 16/35, ADInstruments) and recorded on LabChart software (LabChart v.8, ADInstruments), except oxygen saturation, which was manually inputted as it changed. After instrumentation, blood pressure was recorded in duplicate using an automated blood pressure monitor (M2+, Omron, Kyoto, Japan). A return to flow calibration was performed on the Finometer Pro to derive reconstructed brachial blood pressure; the blood pressure data presented are from the Finometer. Poor beat‐by‐beat blood pressure tracking occurred in three participants, so all blood pressure data presented are based on *n* = 11. $P_{\mathrm{ETC}O_{2}}$, $P_{\mathrm{ET}O_{2}}$, breathing frequency, tidal volume, inspiratory time and CO_2_ production (${\dot{V}}_{CO_{2}}$) were determined breath‐by‐breath via an O_2_/CO_2_ analyser and digital volume transducer (Vyntus CPX, Jaeger Medical, Höchberg, Germany), which was calibrated prior to each participant. The Vyntus system contains an air pressure sensor, and end‐expiratory gases were converted to body temperature, pressure and water vapor saturated partial pressures within the Vyntus system application software (SentrySuite). Minute ventilation (${\dot{V}}_{E}$) was calculated as the product of inspired tidal volume and breathing frequency. Central respiratory drive was estimated by calculating the tidal volume:inspiratory time ratio (*V*_T_:*T*_I_). The ventilatory equivalent for CO_2_ production was calculated as ${\dot{V}}_{E}/{\dot{V}}_{CO_{2}}$. Baseline data were the average of the final minute prior to the first hyperoxia test, and the 1 min prior to the hypoxia test. Facial temperature is reported as the average across the condition (e.g., the entirety of the hyperoxia and hypoxia tests).

#### 2.4.2 Carotid body tonic activity

Chemoreflex‐mediated suppression of ${\dot{V}}_{E}$ was assessed during four 1 min exposures to hyperoxia (100% O_2_; modified Dejours test), which were separated by a minimum of 3 min of room air breathing or until $P_{\mathrm{ET}O_{2}}$ returned to baseline values (Bernards et al., 1966; Gibbons et al., 2022a, 2022b; Prasad et al., 2020). During the room air period, on occasion, investigators would mimic switching the three‐way valve to avoid anticipatory changes in ${\dot{V}}_{E}$ during the hyperoxia (this was avoided in the minute preceding the switch to hyperoxia and during the hyperoxia). The two‐ to three‐breath ${\dot{V}}_{E}$ nadir was noted during the 1 min period once $P_{\mathrm{ET}O_{2}}$ exceeded 250 mmHg, and the average of four trials was used to index carotid body tonic activity. Trials with aberrant breaths or swallows were discarded. The absolute reduction in ventilation during hyperoxia is an indirect estimate of carotid sinus afferent nerve activity, and interpreted herein as carotid body tonic activity, or the carotid body's contribution to eupnoeic ventilation. Cardiovascular parameters (heart rate, blood pressure) were recorded during the final 15 s (whilst $P_{\mathrm{ET}O_{2}}$ was >250 mmHg) of each hyperoxia trial. The change in each parameter (e.g., absolute and relative) is from the 1 min room air breathing immediately prior to each bout of hyperoxia. All data are presented as the average of the four trials. We do not report $P_{\mathrm{ETC}O_{2}}$ during the hyperoxic trials; CO_2_ detection is compromised during periods of 100% O_2_ in metabolic carts that combine CO_2_ and O_2_ analysers in the same system.

#### 2.4.3 Carotid body sensitivity

A poikilocapnic hypoxia exposure was used to assess chemoreflex sensitivity. After $P_{\mathrm{ET}O_{2}}$ returned to baseline from the final hyperoxic bout, the three‐way valve was switched from the hyperoxic Douglas bag to a hypoxic one (9.5% O_2_). After the switch, participants continued to breathe room air for 1 min. Participants breathed from the Douglas bag for 3 min, with the average of the last 30 s recorded for all variables. The change in $P_{\mathrm{ETC}O_{2}}$ per change in $S_{pO_{2}}$ was used as a marker of carotid body sensitivity, with a greater decrease in $P_{\mathrm{ETC}O_{2}}$ representing stronger hypoxic sensitivity and/or weaker breaking action of the hypocapnic feedback control of breathing (Gibbons et al., 2022a, 2022b). During poikilocapnic hypoxia, changes in ${\dot{V}}_{E}$ can be very small; the change in $P_{\mathrm{ETC}O_{2}}$ per change in $S_{pO_{2}}$ is more stable than the change in ${\dot{V}}_{E}$ per change in $S_{pO_{2}}$ and reduces the signal‐to‐noise ratio (Powell, 2007; Steinback & Poulin, 2007). As an additional index of the hypoxic ventilatory response, we also report the change in ventilation versus the change in $S_{pO_{2}}$ (the change in $S_{pO_{2}}$ for this metric is baseline − hypoxia, not hypoxia − baseline; this way the increase in ${\dot{V}}_{E}$ during hypoxia is positive (units: L/min/%) and comparable to previous research; Oeung et al., 2023).

#### 2.4.4 Statistical analysis

All variables were analysed and figures created in RStudio (2025.5.1.513, Posit Software) with R (4.5.1, R Core Team). Linear mixed models (packages *lme4* (Bates et al., 2015), *lmerTest* (Kuznetsova et al., 2017) and *emmeans* (Lenth, 2025)) were performed as: *variable ∼ Condition + TrialOrder +* (*1|Participant*), where *Condition* was facial cooling or thermoneutral, *TrialOrder* was the order of conditions and *1|Participant* was a random intercept for each individual. A significant difference was interpreted when *Condition* had a *P *< 0.05. Normality of residuals was assessed using the Shapiro–Wilk test. When residuals were not normally distributed (*P *< 0.05), data were log‐transformed. When residuals after the log‐transformation remained not normally distributed or when the absolute/relative change variables were not normally distributed, a parametric bootstrap (1000 simulations) for 95% confidence intervals was performed on the non‐transformed model; in these instances, confidence intervals, but not *P*‐values, are presented, and a significant difference is interpreted if the confidence interval did not cross 0. Data from the hyperoxia and hypoxia tests are presented as absolute and relative changes from baseline. To explore the relationship between facial cooling‐induced changes in $P_{\mathrm{ETC}O_{2}}$ and carotid body tonic activity and sensitivity, linear regression analysis was performed. Data are presented as the observed mean ± standard deviation unless otherwise specified. Figures were created using the following packages: *ggplot2* (Wickham, 2016), *ggthemr* (Tobin, 2020), *ggthemes* (Arnold, 2024), *ggpp* (Aphalo, 2025) and *patchwork* (Pedersen, 2025).

## 3 RESULTS

### 3.1 Effects of facial cooling on baseline variables

**FIGURE 2:**
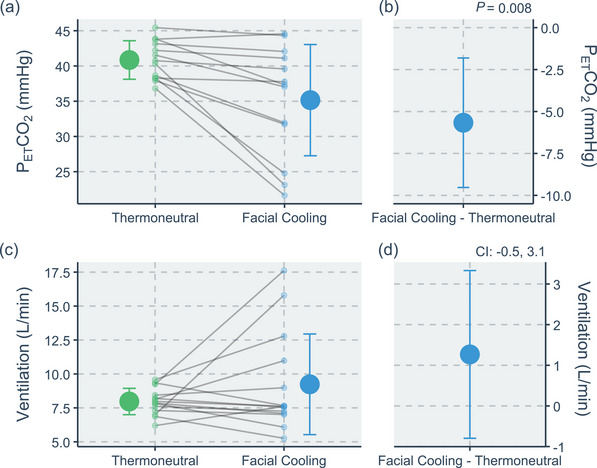
Baseline end‐tidal CO_2_ ($P_{\mathrm{ETC}O_{2}}$) (a, b) and minute ventilation (c, d) during thermoneutral and facial cooling conditions. Panels (a, c) present the observed individual data, means, and standard deviations. Panels (b, d) present the contrast of the estimated marginal means from facial cooling–thermoneutral with 95% confidence intervals (CI). Baseline $P_{\mathrm{ETC}O_{2}}$ was reduced with facial cooling, but minute ventilation was not different. Log‐transformed model residuals were not normally distributed for minute ventilation, so a parametric bootstrap (1000 simulations) for 95% CI was performed on the original model. These do not have *P*‐values; therefore, a significant difference is detected when the CI do not cross 0.

Cheek temperature was 34.2 ± 1.2°C during the thermoneutral condition and dropped to 19.4 ± 3.3°C during facial cooling (*P *< 0.001). Baseline variables from the minute preceding the first hyperoxia trial are presented in Table 1. Blood pressure was markedly elevated with facial cooling, whilst heart rate was unchanged. $P_{\mathrm{ETC}O_{2}}$ was reduced by 5.7 mmHg (95% confidence intervals: −9.5 mmHg, −1.8 mmHg; *P* = 0.008) and ${\dot{V}}_{E}/{\dot{V}}_{CO_{2}}$ elevated by 4.5 (95% confidence intervals: 0.8, 8.1; *P* = 0.02) during facial cooling, whilst no other baseline cardiorespiratory parameters differed. Baseline $P_{\mathrm{ETC}O_{2}}$ and ${\dot{V}}_{E}$ are presented in Figure 2.

**TABLE 1:** Cardiorespiratory parameters at baseline and during hyperoxia.

	Thermoneutral	Facial cooling	*P*
Baseline			
Breathing frequency (breaths/min)	10.3 ± 3	10.7 ± 3.4	0.681
Tidal volume (L)	0.85 ± 0.27	0.91 ± 0.26	0.506^a^
Inspiration time (s)	3.4 ± 1.9	3.1 ± 1.6	CI^b^: −1.4, 1.0
Ventilation (L/min)	8 ± 1	9.2 ± 3.7	CI^b^: −0.5, 3.1
$P_{\mathrm{ETC}O_{2}}$ (mmHg)	41 ± 3	35 ± 8	0.008
$P_{\mathrm{ET}O_{2}}$ (mmHg)	102 ± 5	108 ± 13	0.083
*V*_T_:*T*_I_ (L/s)	0.29 ± 0.06	0.34 ± 0.15	0.635^a^
${\dot{V}}_{CO_{2}}$ (mL/min)	349 ± 55	336 ± 88	0.507
${\dot{V}}_{E}/{\dot{V}}_{CO_{2}}$	23 ± 2	28 ± 6	0.0203
$S_{pO_{2}}$ (%)	99 ± 1	99 ± 1	CI^b^: −0.3, 1.4
Heart rate (beats/min)	56 ± 7	56 ± 6	0.892
Systolic blood pressure (mmHg)	134 ± 10	159 ± 18	0.0004
Diastolic blood pressure (mmHg)	76 ± 8	85 ± 10	0.031
Mean arterial pressure (mmHg)	97 ± 9	113 ± 13	0.005
Hyperoxia			
Breathing frequency (breaths/min)	10.3 ± 2.9	10.9 ± 3.6	0.493
Tidal volume (L)	0.64 ± 0.16	0.68 ± 0.21	0.522
Inspiration time (s)	3.4 ± 1.7	3.3 ± 2	0.790
Ventilation (L/min)	6.1 ± 1	6.7 ± 2.6	CI^b^: −0.9, 1.9
$P_{\mathrm{ET}O_{2}}$ (mmHg)	441 ± 49	422 ± 64	0.549
*V*_T_:*T*_I_ (L/s)	0.23 ± 0.06	0.25 ± 0.12	0.834

Data are presented as mean ± standard deviation. Ventilatory variables are from the 2–3 breath nadir whilst end tidal $P_{O_{2}}$ was >250 mmHg. $P_{\mathrm{ETC}O_{2}}$, $P_{\mathrm{ET}O_{2}}$, ${\dot{V}}_{CO_{2}}$ and ${\dot{V}}_{E}/{\dot{V}}_{CO_{2}}$ are based on *n* = 13. Blood pressures are based on *n* = 11.

^a^ Model residuals were not normally distributed so data were log‐transformed.

^b^ Log‐transformed model residuals were not normally distributed so parametric bootstrap (1000 simulations) for 95% confidence intervals was performed on the original model. These do not have *P*‐values; therefore, a significant difference is detected when the confidence intervals do not cross 0.

### 3.2 Effects of facial cooling on carotid body tonic activity

**FIGURE 3:**
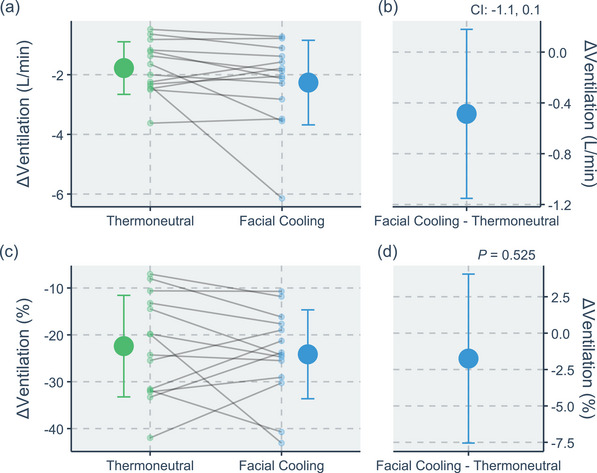
Carotid body tonic activity, presented as the absolute (a, b) and relative (c, d) reduction in minute ventilation during hyperoxia in the thermoneutral and facial cooling conditions. Panels (a, c) present the observed individual data, means and standard deviations. Panels (b, d) present the contrast of the estimated marginal means from facial cooling − thermoneutral with 95% confidence intervals (CI). There was no difference in carotid body tonic activity between conditions. Log‐transformed model residuals were not normally distributed for the absolute reduction in ventilation, so a parametric bootstrap (1000 simulations) for 95% CI was performed on the original model. These do not have *P*‐values; therefore, a significant difference is detected when the CI do not cross 0.

Ventilatory parameters during the 2–3 breath nadir in ${\dot{V}}_{E}$ presented no differences between conditions (Table 1). The absolute and relative changes in cardiorespiratory parameters are presented in Table 2. Carotid body tonic activity, the absolute reduction in ${\dot{V}}_{E}$ during hyperoxia, did not differ (95% CI: −1.1, 0.1); however, the response of all parameters did not differ between facial cooling and thermoneutral. The absolute and relative reduction in ${\dot{V}}_{E}$ during hyperoxia are presented in Figure 3.

**TABLE 2:** Absolute and relative changes in cardiorespiratory parameters during hyperoxia.

	Absolute change			Relative change (%)		
	Thermoneutral	Facial cooling	*P*	Thermoneutral	Facial cooling	*P*
Breathing frequency (breaths/min)	−1 ± 1.2	−0.5 ± 1.2	0.144	−9 ± 10	−5 ± 13	0.205
Tidal volume (L)	−0.11 ± 0.08	−0.16 ± 0.12	0.226	−14 ± 8	−18 ± 13	0.331
Inspiration time (s)	0.5 ± 0.8	0.4 ± 0.7	0.703	21 ± 32	13 ± 20	CI^b^: −25, 10
Ventilation (L/min)	−1.8 ± 0.9	−2.3 ± 1.4	CI^b^: −1.1, 0.1	−22 ± 11	−24 ± 9	0.525
*V*_T_:*T*_I_ (L/s)	−0.07 ± 0.05	−0.09 ± 0.06	CI^b^: −0.05, 0.002	−22 ± 16	−25 ± 14	CI^b^: −10, 5
Heart rate (beats/min)	−2 ± 2	−2 ± 2	0.721	−3 ± 4	−3 ± 4	0.989
Systolic blood pressure (mmHg)	−1 ± 2	−3 ± 6	CI^b^: −5, 2	−1 ± 1	−2 ± 4	CI^b^: −3, 1
Diastolic blood pressure (mmHg)	0 ± 1	−1 ± 3	CI^b^: −3, 1	0 ± 2	−2 ± 4	CI^b^: −4, 1
Mean arterial pressure (mmHg)	−1 ± 1	−2 ± 4	CI^b^: −1, 4	−1 ± 1	−2 ± 4	CI^b^: −4, 1

Data are presented as means ± standard deviation. Ventilatory hyperoxia data are from the 2–3 breath nadir whilst end tidal $P_{O_{2}}$ was >250 mmHg. Cardiovascular hyperoxia data are from the final 15 s of each hyperoxia bout. Blood pressures are based on *n* = 11.

^a^ Model residuals were not normally distributed so data were log‐transformed.

^b^ Log‐transformed model residuals were not normally distributed so parametric bootstrap (1000 simulations) for 95% confidence intervals was performed on the original model. These do not have *P*‐values; therefore, a significant difference is detected when the confidence intervals do not cross 0.

### 3.3 Effects of facial cooling on carotid body sensitivity

**FIGURE 4:**
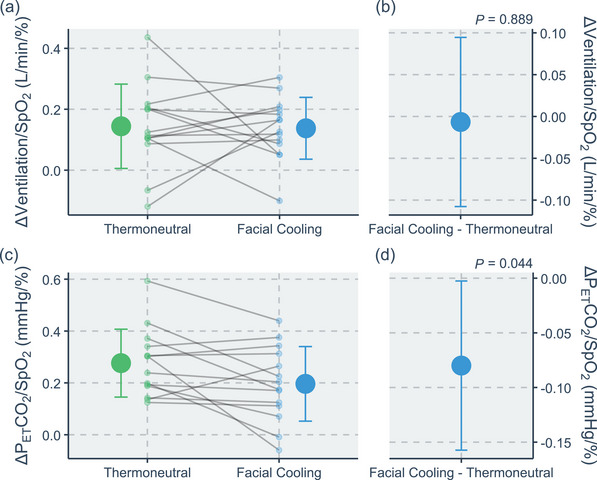
Carotid body sensitivity, presented as the change in minute ventilation (a, b) and end‐tidal CO_2_ per change in oxygen saturation ($P_{\mathrm{ETC}O_{2}}$/$S_{pO_{2}}$) (c, d) during hypoxia (9.5% O_2_) in the thermoneutral and facial cooling conditions. Panels (a, c) present the observed individual data, means and standard deviations. Panels (b, d) present the contrast of the estimated marginal means from facial cooling − thermoneutral with 95% confidence intervals. There was no difference in the change in minute ventilation during hypoxia, but $P_{\mathrm{ETC}O_{2}}$/$S_{pO_{2}}$ was lower with facial cooling.

During hypoxia baseline (Table 3), $P_{\mathrm{ETC}O_{2}}$ remained lower during facial cooling compared to thermoneutral conditions. Mean arterial pressure remained increased, though systolic and diastolic pressures were not. All other parameters did not differ between conditions during the hypoxia baseline. During the last 30s of hypoxia (Table 3), $P_{\mathrm{ETC}O_{2}}$ was lower during facial cooling, as was carotid body sensitivity assessed as $P_{\mathrm{ETC}O_{2}}$/$S_{pO_{2}}$. Table 4 presents the absolute and relative hypoxia‐induced changes in cardiorespiratory parameters. The absolute and relative reductions in $P_{\mathrm{ETC}O_{2}}$ were lower during facial cooling, and the relative increase in tidal volume was lower during facial cooling. All other parameters were not different. The hypoxic ventilatory response, indexed as ${\dot{V}}_{E}$/$S_{pO_{2}}$ and $P_{\mathrm{ETC}O_{2}}$/$S_{pO_{2}}$, is presented in Figure 4.

**TABLE 3:** Cardiorespiratory parameters prior to and during the hypoxia test.

	Thermoneutral	Facial cooling	*P*
Baseline			
Breathing frequency (breaths/min)	12.3 ± 3.7	11.7 ± 2.6	0.427
Tidal volume (L)	0.7 ± 0.16	0.83 ± 0.33	0.059^a^
Inspiration time (s)	2.5 ± 1.1	2.7 ± 1.1	0.447^a^
Ventilation (L/min)	8.1 ± 1.5	8.8 ± 1.9	0.299
$P_{\mathrm{ETC}O_{2}}$ (mmHg)	40 ± 2	37 ± 5	CI^b^: −5.4, −0.7
$P_{\mathrm{ET}O_{2}}$ (mmHg)	106 ± 4	108 ± 7	CI^b^: −3, 6
*V*_T_:*T*_I_ (L/s)	0.31 ± 0.08	0.34 ± 0.08	0.264
${\dot{V}}_{CO_{2}}$ (ml/min)	345 ± 66	342 ± 77	0.9
${\dot{V}}_{E}/{\dot{V}}_{CO_{2}}$	24 ± 3	26 ± 4	0.077^a^
$S_{pO_{2}}$ (%)	99 ± 1	100 ± 1	CI^b^: 0.2, 1.4
Heart rate (beats/min)	55 ± 7	53 ± 8	0.302
Systolic blood pressure (mmHg)	136 ± 10	147 ± 18	0.073
Diastolic blood pressure (mmHg)	75 ± 5	80 ± 11	CI^b^: −1, 10
Mean arterial pressure (mmHg)	98 ± 7	105 ± 13	CI^b^: 1, 15
Hypoxia			
Breathing frequency (breaths/min)	10.7 ± 3.8	11.6 ± 4.8	0.330
Tidal volume (L)	1.06 ± 0.36	1.05 ± 0.53	0.971
Inspiration time (s)	3.4 ± 2.5	3.1 ± 1.7	0.490^a^
Ventilation (L/min)	10.1 ± 1.9	10.2 ± 1.9	0.884
$P_{\mathrm{ETC}O_{2}}$ (mmHg)	37 ± 2	35 ± 4	CI^b^: −3.7, −0.1
$P_{\mathrm{ET}O_{2}}$ (mmHg)	48 ± 5	47 ± 6	0.254
*V*_T_:*T*_I_ (L/s)	0.37 ± 0.1	0.37 ± 0.07	0.949
$S_{pO_{2}}$ (%)	87 ± 4	89 ± 5	0.094
$P_{\mathrm{ETC}O_{2}}$/$S_{pO_{2}}$ (mmHg/%)	0.28 ± 0.13	0.20 ± 0.14	0.044
Ventilation/$S_{pO_{2}}$ (L/min /%)	0.14 ± 0.14	0.14 ± 0.10	0.889

Data are presented as mean ± standard deviation. Hypoxia variables are the average from the final 30 s of poikilocapnic hypoxia (9.5% O_2_). Blood pressures are based on *n* = 11.

^a^ Model residuals were not normally distributed so data were log‐transformed.

^b^ Log‐transformed model residuals were not normally distributed a parametric bootstrap (1000 simulations) for 95% confidence intervals was performed on the original model. These do not have *P*‐values; therefore, a significant difference is detected when the confidence intervals do not cross 0.

**TABLE 4:** Absolute and relative changes in cardiorespiratory parameters during hypoxia.

	Absolute change			Relative change (%)		
	Thermoneutral	Facial cooling	*P*	Thermoneutral	Facial cooling	*P*
Breathing frequency (breaths/min)	−1.6 ± 2	−0.1 ± 3.5	0.141	−13 ± 17	−1 ± 28	0.083
Tidal volume (L)	0.35 ± 0.24	0.22 ± 0.3	0.061	49 ± 30	27 ± 38	0.042
Inspiration time (s)	0.8 ± 1.6	0.4 ± 1.3	CI^b^: −1.1, 0.2	28 ± 45	16 ± 48	0.237
Ventilation (L/min)	2 ± 1.9	1.5 ± 1.2	0.290	27 ± 27	18 ± 13	0.194
$P_{\mathrm{ETC}O_{2}}$ (mmHg)	−3.1 ± 1.2	−1.9 ± 1.6	0.027	−8 ± 3	−5 ± 4	0.042
$P_{\mathrm{ET}O_{2}}$ (mmHg)	−58 ± 5	−61 ± 9	0.280	−55 ± 4	−56 ± 6	0.201
*V*_T_:*T*_I_ (L/s)	0.06 ± 0.09	0.03 ± 0.06	0.228	23 ± 37	12 ± 19	0.168
$S_{pO_{2}}$ (%)	−12 ± 4	−11 ± 5	0.258	−12 ± 4	−11 ± 5	0.239
Heart rate (beats/min)	10 ± 8	11 ± 8	0.588	20 ± 16	22 ± 15	0.579
Systolic blood pressure (mmHg)	−1 ± 3	−8 ± 13	CI^b^: −14, 2	−1 ± 2	−5 ± 8	0.129
Diastolic blood pressure (mmHg)	0 ± 3	−3 ± 7	0.214	−1 ± 4	−4 ± 7	0.244
Mean arterial pressure (mmHg)	−1 ± 4	−5 ± 8	0.130	−1 ± 4	−5 ± 7	0.153

Data are presented as mean ± standard deviation. Data are the change from the 1‐min baseline prior to hypoxia to the average of the final 30s of the 3‐min bout of poikilocapnic hypoxia (9.5% O_2_). Blood pressures are based on *n* = 11.

^a^Model residuals were not normally distributed so data were log‐transformed.

^b^ Log‐transformed model residuals were not normally distributed so parametric bootstrap (1000 simulations) for 95% confidence intervals was performed on the original model. These do not have *P*‐values; therefore, a significant difference is detected when the confidence intervals do not cross 0.

### 3.4 Relationship between facial cooling‐induced change in $P_{\mathrm{ETC}O_{2}}$ and carotid body function

**FIGURE 5:**
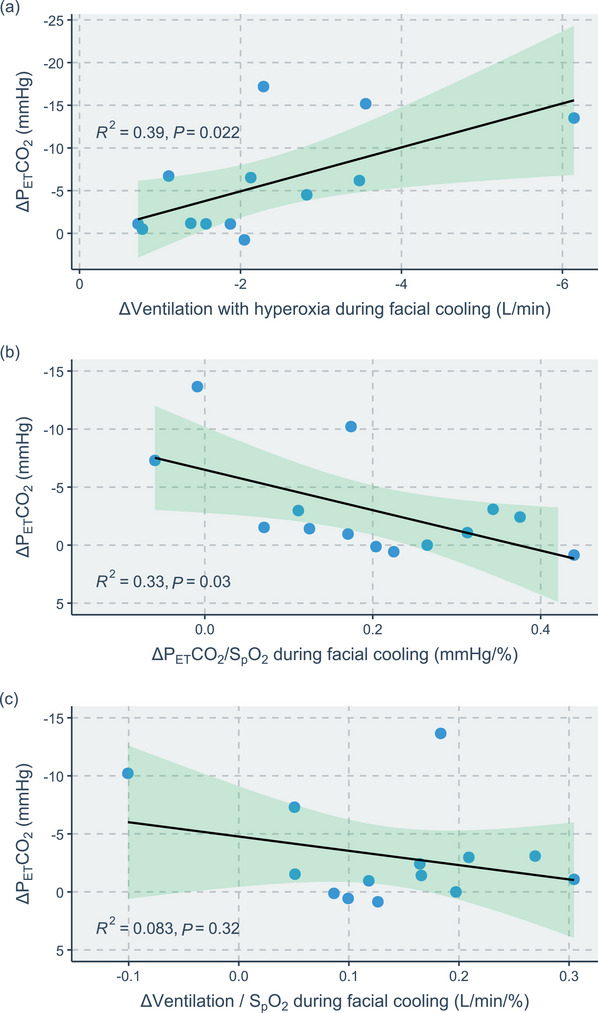
Relationships between the change in baseline end‐tidal CO_2_ ($P_{\mathrm{ETC}O_{2}}$) during facial cooling (facial cooling $P_{\mathrm{ETC}O_{2}}$ − thermoneutral $P_{\mathrm{ETC}O_{2}}$) and carotid body tonic activity (the reduction in minute ventilation during hyperoxia) (a), carotid body sensitivity (the change in $P_{\mathrm{ETC}O_{2}}$ per change in oxygen saturation ($S_{pO_{2}}$) during hypoxia) (b), and the hypoxic ventilatory response (the change in minute ventilation per change in $S_{pO_{2}}$ during hypoxia; note this $S_{pO_{2}}$ is calculated as baseline − hypoxia in order to yield a positive value, that is increase in ventilation per drop in $S_{pO_{2}}$, in line with previous research) (c). The change in $P_{\mathrm{ETC}O_{2}}$ was from the baseline preceding that test (e.g., hyperoxia baseline in panel (a) and hypoxia baseline in panels (b, c)). Facial cooling‐induced hyperventilation was related to carotid body tonic activity and sensitivity, but not the hypoxic ventilatory response. Note that the *y*‐axes are inverted to depict facial‐cooling‐induced hyperventilation.

The change in $P_{\mathrm{ETC}O_{2}}$ during facial cooling (e.g., the baseline before hyperoxia) was related to the absolute reduction in ${\dot{V}}_{E}$ during hyperoxia during facial cooling (Figure 5a; *R* ^2^ = 0.39, *P* = 0.022). Further, a change in $P_{\mathrm{ETC}O_{2}}$ during facial cooling (e.g., the baseline before hypoxia) was inversely related to carotid body sensitivity during facial cooling (Figure 5b; *R* ^2^ = 0.33, *P* = 0.03); however, this relationship needs to be taken with caution, given the influence of mathematical coupling. By contrast, the change in $P_{\mathrm{ETC}O_{2}}$ during facial cooling during hypoxia baseline was not related to the hypoxic ventilatory response indexed as ${\dot{V}}_{E}$/$S_{pO_{2}}$ during facial cooling (Figure 5c; *R* ^2^ = 0.0052, *P* = 0.82).

## 4 DISCUSSION

We investigated the effect of facial cooling on carotid body tonic activity and sensitivity. Facial cooling (i) caused hyperventilation at rest; (ii) did not alter carotid body tonic activity; and (iii) reduced carotid body sensitivity to poikilocapnic hypoxia; and (iv) the induced hyperventilation was related to increased carotid body tonic activity. These findings suggest that trigeminal nerve afferent stimulation augments ventilatory drive through mechanisms that are at least partially independent of carotid body chemoreflex pathways.

### 4.1 Facial cooling increases ventilation

The cold shock response is characterised by sympathetically mediated tachycardia, uncontrollable hyperventilation, peripheral vasoconstriction and hypertension mediated by cutaneous thermoreceptors (Tipton, 1989). Consistent with this response, we observed increased blood pressure, ${\dot{V}}_{E}/{\dot{V}}_{CO_{2}}$ and reduced eupnoeic $P_{\mathrm{ETC}O_{2}}$ with facial cooling. These findings also align with previous reports of whole‐body cold‐induced hyperventilation (Datta & Tipton, 2006; Duffin et al., 1975; Gibbons et al., 2022b) and facial cooling‐induced hyperventilation (Jay et al., 2007; Stewart et al., 1998). We did not observe an increase in heart rate, likely due to concurrent parasympathetic activation by trigeminal nerve stimulation and the mammalian dive reflex (Finley et al., 1979). Facial cooling, independent of whole‐body cooling, is also a potent sympathetic nervous system stimulus (Fagius & Sundlöf, 1986; Fisher et al., 2015; Heindl et al., 2004). Indeed, cooling the forehead increases sympathetic nerve activity more than cooling the hand (Heindl et al., 2004), highlighting the sensitivity of trigeminally innervated facial regions. Furthermore, the face has greater alliesthesial thermosensitivity compared to other skin regions, resulting in enhanced cooling‐induced discomfort (Cotter & Taylor, 2005). Together, these responses reinforce the notion that isolated facial cooling, through both thermal discomfort and trigeminal activation, can elicit a robust ventilatory response, mirroring key features of the cold shock response even in the absence of whole‐body cooling.

Contrary to our findings, previous studies have not observed reductions in $P_{\mathrm{ETC}O_{2}}$ during facial cooling (AlSalahi et al., 2020; Argacha et al., 2008; Mukhtar & Patrick, 1986). This may in part be due to those studies not exposing the eyes to the cold (AlSalahi et al., 2020; Argacha et al., 2008; Mukhtar & Patrick, 1986). We intentionally maximised trigeminal nerve stimulation by cooling the entire face and eyes; our findings agree with facial immersion studies, which show reductions in $P_{\mathrm{ETC}O_{2}}$ when the face is immersed in 0°C water, but not 10°C (Jay et al., 2007). Alternatively, there may be considerable heterogeneity in ventilatory responses to facial cooling, as evident from our sample, and consistent with studies on ventilatory responses to thermal stress (Gibbons et al., 2022a, 2022b). Although elevated carotid body tonic activity may have contributed to the reductions in $P_{\mathrm{ETC}O_{2}}$ (see below), trigeminal nerve afferents and sympathetic inputs converge on brainstem regions (e.g., nucleus tractus solitarius; Boscan & Paton, 2002; Marfurt & Rajchert, 1991; Zerari‐Mailly et al., 2005), likely contributing to increased ventilation independent from the carotid body.

### 4.2 Facial cooling and carotid body function

We conducted this study to understand how facial cooling might increase the poikilocapnic hypoxic ventilatory response (Argacha et al., 2008). We speculated that facial cooling would induce carotid body hyperexcitability, akin to moderate whole body cooling (Gibbons et al., 2022b). By contrast, we observed baseline hyperventilation with facial cooling, and whilst this was related to increases in carotid body tonic activity, we observed a subtle effect (facial cooling − thermoneutral 95% CI: −1.1, 0.1 L ΔL/min) of facial cooling on hyperoxic ventilatory suppression. This hypocapnia persisted throughout the trial and likely contributed to the reduction in hypoxic sensitivity (e.g., Figure 5b), as measured by the $P_{\mathrm{ETC}O_{2}}$/$S_{pO_{2}}$ (Blain et al., 2009; Smith et al., 2015). This hypocapnic restraint on the hypoxic ventilatory response is well‐established (Corne et al., 2003). However, unlike the hyperoxic suppression in ventilation, the facial cooling‐induced reduction in $P_{\mathrm{ETC}O_{2}}$ was not related to the hypoxic ventilatory response. This is consistent with findings from passive and active heat‐induced hyperventilation (Gibbons et al., 2022a, 2022b), which emphasise the distinction between the hyperoxia and hypoxic tests, and how the ventilatory suppression during hyperoxia better explains alterations in carotid body function relevant to changes in room air breathing.

### 4.3 Implications

Our findings highlight the overlap between the cold shock response and intense facial cooling. Facial cooling in isolation frequently occurs during outdoor activities in cold conditions, and is amplified when combined with high wind speeds or water exposure (Molnar, 1946; Tikuisis & Osczevski, 2003). In these instances, frostbite of the face is common (Harirchi et al., 2005; Hassi & Mäkinen, 2000; Lehmuskallio et al., 1995). Moreover, cold temperatures and heavy snowfall increase the risk of myocardial infarction (Auger et al., 2017) and this is often attributed to exertional work such as shovelling. However, pre‐existing risk factors do not seem to have independent associations with snow‐shovelling‐related acute coronary syndromes, suggesting other factors may increase susceptibility (Nichols et al., 2012). A brisk ventilatory response to facial cooling may reduce myocardial blood flow and oxygen supply (Neill & Hattenhauer, 1975; Rowe et al., 1962; Yokoyama et al., 2008), potentially secondary to hypocapnia‐induced vasoconstriction (Case et al., 1978). Extreme cold and snowfall are also independent risk factors for death from haemorrhagic stroke in men (Polcaro‐Pichet et al., 2019). Hypocapnia constricts the cerebral vasculature (Wei et al., 1980), leading to reductions in brain blood flow (Hoiland et al., 2019). Thus, combined with its sustained cold shock pressor response (Schlader et al., 2016), facial cooling‐associated hyperventilation may meaningfully contribute to increased risk of shovelling‐related myocardial infarctions and stroke.

The physiological stress modelled in this study reflects that experienced during high‐altitude mountaineering and many other winter activities, where the body is well insulated, but the face remains exposed to extreme cold. A similar pattern occurs in cold‐water swimming or surfing, when wearing wetsuits, only the face is exposed to cold water and air, resulting in intense facial cooling. In these contexts, facial cooling, combined with hypoxia (as is often experienced during mountaineering), may reduce cerebral oxygen delivery and reaction time as previously observed with whole body cooling (Gibbons et al., 2020).

### 4.4 Limitations and future directions

We did not assess ventilatory responses to isocapnic hypoxia, which would have helped to comprehensively characterise carotid body function (Gibbons et al., 2022b) and aid with the interpretation of carotid body sensitivity under conditions of different baseline $P_{\mathrm{ETC}O_{2}}$. Our concern was a potential decay in the response to facial cooling; indeed, although $P_{\mathrm{ETC}O_{2}}$ remained reduced and blood pressure elevated during the hypoxia baseline period, the magnitude was lower than the hyperoxia baseline. Alternatively, measuring carotid body sensitivity via transient hypercapnia (McClean et al., 1988) would have complemented measures of tonic activity and provided additional insight into the effects of facial cooling on carotid body function. Additionally, we did not test enough females to look at the effect of sex; however, the three females who participated did not respond differently. Although elucidating the effects of heightened blood pressure and lowered $P_{\mathrm{ETC}O_{2}}$ on cerebral blood flow would have provided additional insight, we did not want to interfere with ventilatory responses. Lastly, there were no apparent reasons for the heterogeneity in the ventilatory response. This heterogeneity may have been influenced by psychophysiological factors, rather than purely cold shock response or trigeminal nerve stimulation, since anxiety increases the magnitude and duration of hyperventilation during cold water immersion (Barwood et al., 2013). These complications were anticipated; however, we opted for ecological validity in our study design to understand how ventilation is manipulated under severe facial cooling. To isolate the interaction between trigeminal nerve stimulation and carotid body function, future studies should consider non‐thermal trigeminal nerve activation to mitigate the confounding cold shock response and psychophysiological contributions of physical facial cooling. Hence, it removes the psychophysiological contribution to ventilatory changes and better demonstrates the impact of cooling on the carotid body. Alternative methods could include the use of menthol, which chemically activates the transient receptor potential melastatin 8 channel (Peier et al., 2002) without cooling the skin (Lasanen et al., 2016). Inhaled menthol and menthol‐containing chewing gum decrease the perception of breathlessness (Aucoin et al., 2023); however, this has not been applied to the face to assess trigeminal nerve stimulation or subsequent influence on ventilation.

### 4.5 Conclusion

Facial cooling, an ecologically relevant and potent trigeminal nerve stimulus, provoked hyperventilation and sustained reductions in $P_{\mathrm{ETC}O_{2}}$. These reductions in $P_{\mathrm{ETC}O_{2}}$ were likely related to increases in carotid body tonic activity and blunted hypoxic sensitivity. Although there may have been a subtle increase in carotid body tonic activity, this is unlikely to explain the observed baseline hyperventilation. Facial cooling does not induce carotid body hyperexcitability. We interpret these findings to suggest that facial cooling‐induced hyperventilation resembles the cold shock response and is likely mediated by trigeminal nerve‐driven sympathoexcitation originating from skin cold thermoreceptors. This has implications for understanding individual variability in ventilatory responses to cold and potential cardiovascular risks in cold environments.

## AUTHOR CONTRIBUTIONS

This study was conducted in the physiology laboratory in the Cardiff School of Sport and Health Sciences (Cyncoed Campus) at Cardiff Metropolitan University. *Conception or design of the work*: Robyn Morley and Joshua C. Tremblay. All the authors worked on the acquisition, analysis or interpretation of data for the work, drafting the work or revising it critically for important intellectual content. All authors approved the final version of the manuscript; agree to be accountable for all aspects of the work in ensuring that questions related to the accuracy or integrity of any part of the work are appropriately investigated and resolved; and all persons designated as authors qualify for authorship, and all those who qualify for authorship are listed.

## CONFLICT OF INTEREST

The authors have no conflicts of interest to declare.

## FUNDING INFORMATION

None.

## Data Availability

The data that support the findings of this study are available from the corresponding author upon reasonable request.
